# The Effect of Parental Caregiving on the Fertility Expectations of Adult Children

**DOI:** 10.1007/s10680-024-09724-4

**Published:** 2024-11-27

**Authors:** Ester Lazzari, Valeria Zurla

**Affiliations:** 1 Wittgenstein Centre for Demography and Global Human Capital, IIASA, OeAW, University of Vienna, Vienna, Austria; 2 Center for Studies in Economics and Finance, University of Naples Federico II, Naples, Italy

**Keywords:** Fertility expectations, Parental caregiving, Intergenerational relations Australia

## Abstract

Previous research has highlighted the positive impact of parents on their adult children's fertility plans through childcare, but the association between parental health and fertility expectations remains unclear. Thus, this paper offers a novel perspective on the issue of family support by investigating how caregiving responsibilities toward elderly parents affect adult children’s decision to have a child. Using a long panel dataset for Australia, we examine whether adult children changed their fertility expectations after becoming care providers to their parents. To address issues of unobserved heterogeneity and selection into parenthood and caregiving, we employ generalized difference-in-differences models. Results show a 7% decrease in fertility expectations within two years of becoming a parental caregiver, with a stronger effect over time, consistent across genders and more pronounced for respondents with one child. These findings suggest that interventions aimed at reducing the caregiver burden could provide an opportunity to positively influence fertility levels.

**Supplementary Information:**

The online version contains supplementary material available at 10.1007/s10680-024-09724-4.

## Introduction

The physical and emotional demands of caregiving can be substantial. This impact extends not only to the elderly person in need of care but also to their adult children, who often step in to provide care and support. Parental caregiving can be emotionally overwhelming and stressful, particularly if it requires a significant time commitment or if the parent has a serious health condition (Fortinsky et al., 2007; Schulz et al., 2020). It also entails significant financial, social, and psychological costs and it can be detrimental for individuals’ overall wellbeing (Al-Janabi et al., 2019; Labbas & Stanfors, 2023; Van Houtven et al., 2019). Despite these challenges, parental caregiving can also be an expression of love that can be fulfilling for many (Fingerman et al., 2007). This ambivalence is a typical feature of the relation between older adults and their adult children (Connidis & McMullin, 2002; Luescher & Pillemer, 1998) and can be particularly strong when balancing caregiving with other significant life transitions (Elder, 1995). Therefore, a decline in parental health may have far-reaching consequences on a number of life choices and expectations about the future of adult children, including their fertility plans. They may face difficult choices and trade-offs between caring for their aging parents and starting a family of their own. Against this background, this study aims to investigate whether and how parental caregiving responsibilities affect the fertility preferences of adult children.

The prevalence of chronic health conditions and disability increases with age. Hence, caregiving responsibilities toward aging parents may become an increasingly relevant barrier to forming a family as adult children move through the life course and may especially hinder the fulfillment of fertility plans for those who have postponed family formation. Additionally, the added stress of being a caregiver may arise unexpectedly, such as in the case of a sudden deterioration in parental health. Fertility plans may be difficult to readjust in response to such unexpected shifts, potentially resulting in a long-lasting impact on an individual’s ability to form a family. This effect may be especially pronounced for those who have delayed parenthood and have less time left to make this life transition. This issue is further compounded by the significant ongoing delay in fertility observed since the 1970s (Beaujouan, 2020; Prioux, 2005). As a result, caregiving responsibilities may eventually have a real impact on childbearing recuperation and fertility levels in countries where fertility has been postponed. However, whether older people represent an increasing challenge to family formation or a source of support for the middle generation ultimately depends on their status of health. For instance, a study by Margolis and Wright (2017), contributing to the ongoing and broader debate about the relationship between longevity and morbidity (Crimmins et al., 2009; Permanyer & Bramajo, 2023), shows that, despite fertility postponement, the period during which grandparents overlap with their grandchildren while remaining in good health is increasing in the USA due to improvements in health at older ages.

Theoretically, a deterioration in parental health may negatively impact the childbearing plans of adult children in two ways. First, through the time and energy devoted to caregiving, leaving fewer resources for other activities such as starting a family; and second, by disrupting the provision of informal childcare. In Western high-income countries, there is a substantial involvement of grandparents in childcare, with most countries seeing more than half of grandparents actively contributing to the care of their grandchildren (Baxter, 2022; Bordone et al., 2017; Di Gessa et al., 2020; Glaser et al., 2014). Providing this type of assistance could prove challenging as they experience declining health, resulting in their adult children having to depend more on paid childcare services instead of the support provided by grandparents.

The literature so far has focused on the positive impact that grandparents have on the realized and intended fertility of their adult children through the provision of different forms of support, particularly childcare (i.e., Aassve et al., 2012a; Rutigliano, 2024; Tanskanen & Danielsbacka, 2021; Yoon, 2017). Okun and Stecklov (2021) drew attention to the perspective of the adult child and demonstrated how parental loss, another key feature of the intergenerational relationship between parents and children, negatively impact the fertility of adult children in Israel. However, little attention has been given to the effects of parental care needs on reproductive outcomes and it remains unclear whether the declining health of elderly parents is related to the revision of the fertility expectations of their adult children later in life. This is despite the fact that it is a prominent feature of intergenerational linkages, and with a potentially increasing impact on fertility due to the changing demographics mentioned above. Hence, this paper offers a novel perspective on the issue of family support, with the adult children being the providers, rather than the recipients, of support.

We use a long panel of survey data representative of the entire adult Australian population from 2001–2021 and examine whether prospective parents changed their fertility expectations after becoming care providers to their parents. To address issues of unobserved heterogeneity and selection into parenthood and caregiving, we employ a generalized difference-in-differences model. This approach allows us to compare the changes in fertility expectations before and after the event of becoming a caregiver, and then compare these changes to those of control individuals that did not become caregivers. The results provide important information about the relationship between parental health and childbearing intentions and contribute to a deeper understanding of the complex multigenerational processes influencing individuals’ fertility expectations.

## Parental Influence on Adult Children’s Fertility Choices

Demographic changes are altering the life course context in which individuals become parents. Because of childbearing postponement, the transition into the grandparent role has been delayed across cohorts (Leopold & Skopek, 2015; Margolis, 2016). Since health deteriorates with age, an increasing share of prospective grandparents may thus be in need of care rather than able to provide care by the time their children start planning to have a child of their own. Despite the increase in the number of potential helpers (Perry & Daly, 2017), grandparents remain the most common providers of childcare (Baxter, 2022; Sear & Coall, 2011), which suggests that an alteration in the flow of support from grandparents can have important implications for fertility expectations. In this section, we detail the pathways through which caregiving responsibilities toward parents may affect the fertility expectations of their adult children.

First, research has examined how in low-fertility settings grandparents have the potential to influence decisions regarding the family size of their adult children through intergenerational transfers. This is based on the idea that grandparents provide different types of support, including emotional, financial, and time-based resources, which can mitigate the perceived costs of parenthood (Aassve et al., 2012a; Harknett et al., 2014; Rutigliano, 2020). Consequently, these forms of assistance can positively affect an individual’s decisions regarding having children and ultimately lead to higher fertility rates.

Several studies have presented empirical evidence that grandparental support leads to higher childbearing intentions among adult children (Rutigliano & Lozano, 2022; Tanskanen & Danielsbacka, 2021; Tanskanen & Rotkirch, 2014) and facilitates the transition into parenthood (Rutigliano, 2020). Additionally, research has shown that parental support increases the likelihood of having a second or higher order birth (Aassve et al., 2012b; Rutigliano, 2020; Yoon, 2017). Our study relates to this body of literature because it recognizes the value of intergenerational linkages that tie grandparents to the fertility choices of their adult children. In particular, we posit that grandparents who are unwell or have health issues will be less able to provide childcare, and hence may cause a decline in the fertility expectations of their adult children through the loss of this important potential source of support. In their estimation of the impact of parental death on the fertility of adult children, Okun and Stecklov (2021) bolster the idea that a loss of grandparental childcare services due to grandparental death may indeed influence a downward revision of fertility expectations. Clearly, market-based or government-funded childcare options can also allow families to delegate some of their childcare responsibilities to others (Di Gessa et al., 2016). However, grandparental childcare is often viewed as the ‘best’ form of childcare there is (Wheelock & Jones, 2002), as it is a more reliable and cost-effective alternative, and it plays a more important role in mitigating parenting stress for both mothers and fathers than any other form of childcare (Craig & Churchill, 2018).

Second, adult children who are responsible for caring for their elderly parents or parents-in-law may face challenges in balancing their caregiving responsibilities with having children of their own due to a lack of time, energy, or other resources. For instance, they may experience a reduction in their labour force participation and earnings, especially in countries which do not provide sufficient opportunities to deal with adverse health events of family members (Frimmel et al., 2020; Hammer & Neal, 2008; Løken et al., 2017; Reelstab et al., 2020; Vangen, 2021). Caring for older parents can also be emotionally demanding and have implications on mental health and wellbeing (Fortinsky et al., 2007; Hammer & Neal, 2008; Schulz et al., 2020), which may also impact fertility expectations (Lawley et al., 2022). As parental health declines, adult children may anticipate the risk of being ‘sandwiched’ between the needs of their future children and their parents (Hammer & Neal, 2008; Perrig-Chiello & Hopflinger, 2005). This new source of uncertainty can lead to a decrease in perceived behavioral control, which may negatively affect their attitudes and intentions toward having children (Ajzen, 1991).

The empirical literature reviewed above indicates that an increase in caregiving responsibilities toward elderly parents may be associated with a decreased expectation for having children due to both a lack of childcare and resources available to raise children. Aside from these material resources, psychological sentiments may also play a role. Specifically, providing care for aging parents may increase the value that adult children place on family and intergenerational relationships. As a result, they may feel a stronger desire to have children of their own or may perceive a need to accelerate fertility, both as a way of continuing the family lineage and ensuring that their parents can witness the birth of their grandchild. This theoretical argument is consistent with empirical research suggesting that caregiving can lead to positive outcomes for the caregiver, including increased feelings of purpose and meaning in life (Cohen et al., 2002; Mendez-Luck et al., 2008). It is also consistent with the finding that familial death can prompt changes in psychological understandings in a way that positively influences fertility plans (Rackin & Gibson-Davis, 2022). Hence, the effect of a decline in parental health on the fertility expectations of their children is theoretically unclear and requires empirical investigation.

## Research Context

Research has found a clear association between the policy environment and the frequency of grandparental childcare: in countries with less generous childcare services parents receive more frequent childcare help from grandparents (Bordone et al., 2017; Di Gessa et al., 2016). In Australia, the childcare system is mainly market-driven and receives government fundings through the Child Care Subsidy (Gray et al., 2022). Despite this support, the costs of childcare remain relatively high compared to other wealthy societies. For example, a family with two children, where both parents work full-time, spends around 26% of their average earnings on childcare, whereas the OECD average is 17% (OECD, 2019). At the same time, a strong social expectation to re-enter the workforce after childbirth has been documented. For instance, findings from a national survey of Australian women indicate that when asked about future aspirations in their early 20s, only 5% of women aspired to work unpaid at home (Johnstone & Lee, 2009). Despite this situation, the employment rate for Australian women aged 15–64 with at least one child under the age of 15 is 69%, which is below the OECD average and lower than comparable countries like the UK and New Zealand (OECD, 2019).

Considering the challenging landscape of childcare costs and the societal emphasis on women re-joining the workforce, it is likely that, similarly to what observed for England (Di Gessa et al., 2020; Kanji, 2018), informal childcare plays an important role in facilitating the balance between work and family life for Australian women. Historically and to this day, informal care has been primarily provided by grandparents and it plays a key role in Australia’s childcare landscape (Baxter, 2013), with almost two-thirds of Australian grandparents providing some form of childcare (Baxter, 2022).

Similarly, Australia’s aged care system only partially supports families in caring for older generations. Since the 1980s, Australian governments have prioritized home-based care over institutional settings, in line with older Australians’ preference to remain in their communities for as long as possible. This shift has been facilitated through a range of home-based services provided by the government, supplemented by significant reliance on familial care (Yeandle & Kröger, 2013). The Australian Government provides funding for services which support frail older people to enhance their independence and prevent their premature admission to long-term residential care through the Home Care Packages Program (Australian Government Department of Health, 2023). The success of these initiatives heavily relies on support from family members, predominantly adult children (mainly daughters), motivated by family responsibility, emotional obligation, and the belief that they can provide better care than anybody else ( Australian Bureau of Statistics, 2018). These family members play a crucial role in organizing and managing health and social care for their parents and often provide much of this care themselves (Yeandle & Cass, 2013). Due to prolonged waiting times for home care packages, the presence of a family member to fill the gap is often necessary (Australian Institute of Health and Welfare, 2023a). Financial assistance is available through income support payments for those unable to work due to caregiving responsibilities, though only a small subset of informal carers receive it. Additionally, the Carer Gateway program offers free services to support carers, such as peer support groups, online skill courses, counselling, and access to emergency respite (Australian Institute of Health and Welfare, 2023b). Carers in Australia also have the legal right to request flexible working arrangements if they have worked for the same employer for at least one year. Despite these programs, research indicates that many carers still have unmet needs for additional financial, physical, and emotional support (Temple & Dow, 2018).

Recent estimates indicate that around 1 in 10 adults in Australia provide unpaid care to an aging or disabled family member (Australian Institute of Health and Welfare, 2021a). The likelihood of needing to provide care for aging parents increases over the course of the reproductive life, as the prevalence of adverse parental health conditions tends to rise with age. In fact, the proportion of people aged 25 to 34 who receive public financial support for their caregiving tasks (i.e., Carer Allowance) is 9.9%, while this figure is almost double for those in the 35 to 44 age category (Australian Institute of Health and Welfare, 2021b). Interestingly, 22% of reproductive-age men and women consider caring responsibilities (e.g., for those with a disability or elderly) to be a crucial factor in their decision about whether to have a child in the future (Gray et al., 2022).

Fertility postponement represents another major demographic shift in Australian demography (Lazzari, 2021a). Since the mid-1907s, the median age of mothers at childbirth has increased by 6 years, rising from 25.8 to 31.9, while the median age of fathers has increased by 5 years, from 28.6 to 33.7 (Australian Bureau of Statistics, 2022). Typically, parenthood begins in the late 20s to early 30s and is followed by a period of intensive childbearing. This shift toward later childbearing has corresponded with a general decline in the completed family size of successive cohorts of women (Lazzari, 2021b). Despite these trends, the social norm of having two children remains prevalent in Australia, with over 80% of women transitioning to a second child after the first (Gray & Lazzari, 2023).

## Data and Methods

### Data

The analyses draw on data from the Household Income and Labour Dynamics in Australia (HILDA) Survey, a nationally representative panel study annually undertaken in Australia since 2001 through a combination of self-completion questionnaires and face-to-face interviews (Wooden & Watson, 2002). The HILDA dataset contains detailed information on a wide range of aspects of life, including desires and expectations to have children, and caregiving responsibilities toward the elderly.

Because HILDA started to collect data on caregivers only from wave 5, the first four waves of data are excluded from our analysis. In addition, we do not use data collected in waves 5, 8,11,15, and 19 because in these waves different filters are applied to determine who can answer questions related to childbearing desires and expectations. Our final sample totalled 1,559 individuals aged 18 to 49.

### Dependent and Independent Variables

Our analysis focuses on expected fertility, which refers to the number of children people anticipate having, as opposed to their intended or desired family size. Fertility desires reflect individuals’ ideal plans and are influenced by perceptions formed in young adulthood of what an ideal family should be (Rackin & Bachrach, 2016). Intentions consider the practical constraints individual face in achieving their reproductive goals (Ajzen & Klobas, 2013; Miller & Pasta, 1993, 1995). Consequently, intentions can change over time, adapting to the opportunities and constraints of having a child, while desires are thought to remain relatively stable and less affected by changing circumstances. Expectations are empirically similar to intentions (Morgan 2011), as they also consider external constraints that are beyond an individual’s direct control (Iacovou & Tavares, 2011). However, they differ in that they also include external factors beyond the individual’s direct control, such as parental health, which can influence the realization of childbearing plans. As such, expectations are more strongly associated with fertility behaviors than desires or intentions. Previous studies using HILDA (Drago et al., 2011; Fan & Maitra, 2013) have found that fertility expectations are strongly predictive of realized fertility one year later. Given our interest in how parental health, an external factor, affects considerations of having children, this study focuses on expectations.

We measure fertility expectations, the dependent variable, using responses to the question: “How likely are you to have more children in the future?”, which ranges from 0 (Very unlikely) to 10 (Very likely). For our regression analyses, we treat this variable as continuous.

The main exposure variable of interest is the respondent caregiver’s status. Caregivers are identified within the HILDA survey by asking respondents if they provide ongoing care or help with activities of daily living to a family member who has a long-term health condition, is elderly, or has a disability. Our analysis focuses specifically on individuals who provide informal care to a parent or parent-in-law.

To examine the effect of caregiving on fertility expectations, we focus on cases where the need for parental care arises at a time when respondents have an expectation of having a child in the future. Specifically, we restrict the sample to individuals who, prior to becoming caregivers, expressed an interest in having children (i.e., provided a score of 4 or higher on the fertility expectations scale). By doing so, we ensure that the analysis includes only individuals with an actual interest in having children. These are childless individuals wishing to become parents or parents with one or more children wishing to have another child. For this subset, caregiving responsibilities could disrupt their fertility expectations compared to those who do not anticipate having a child, and hence have repercussions on fertility outcomes.

A vast body of literature has used HILDA to investigate different features of childbearing goals among Australian men and women, highlighting influence of factors such as age, relationship status, employment conditions, parity, and health status (Beaujouan, 2019; Gray et al., 2013; Gray et al., 2022; Lazzari et al., 2023; Testa and Bolano, 2021). Therefore, in our analyses, we include the following time-variant variables as controls: marital status (married, cohabiting, single), employment status (employed, unemployed, outside the labour force), age, and number of children ever born.

**Fig. 1:**
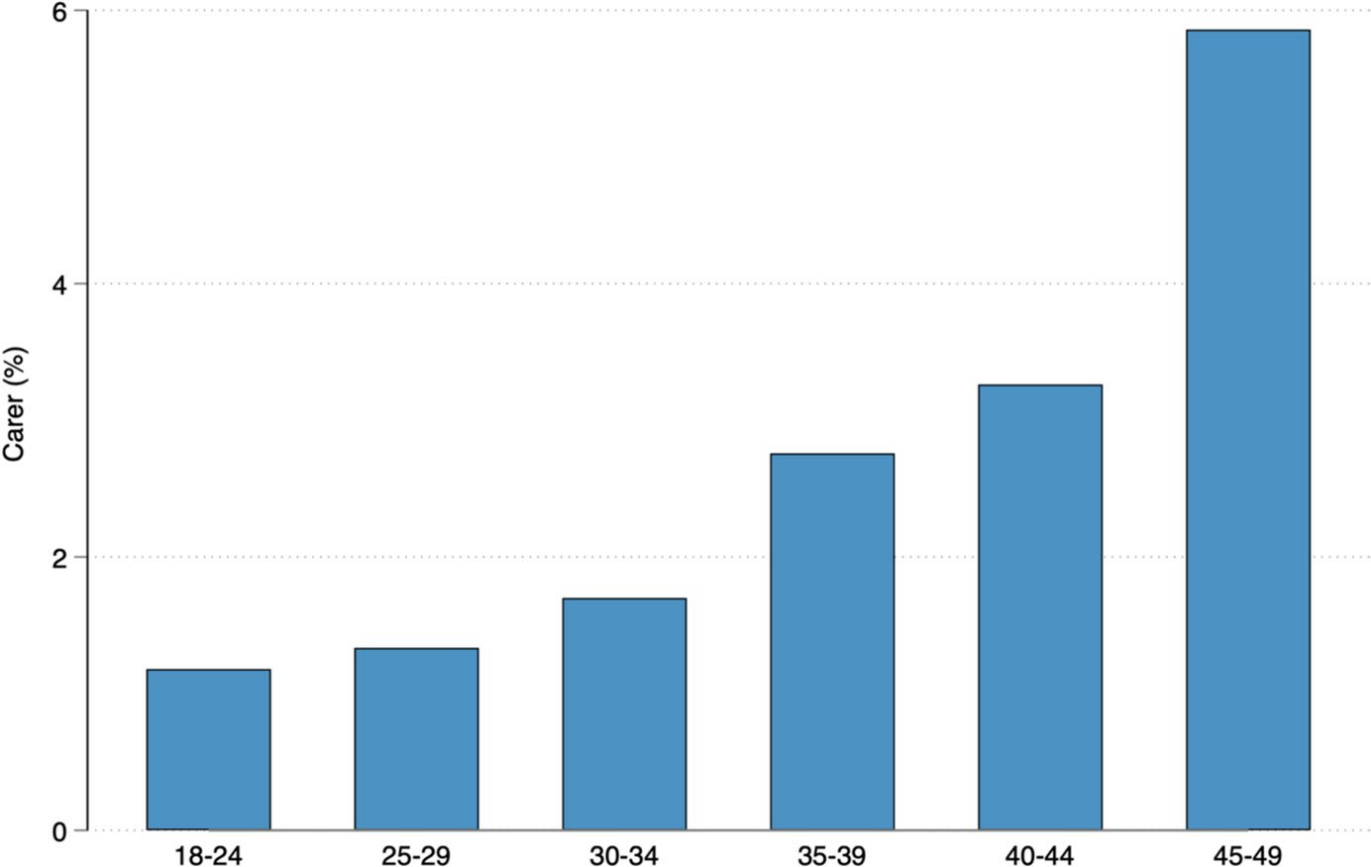
Informal carers of a parent or parent-in-law by age group (weighted). *Source*: HILDA, waves 6–21, release 21

Table 1 presents summary statistics for all variables analyzed in this study. The average age of the adult children included in the sample was 29.6 years, with a majority being childless (62%) and slightly over half married or cohabiting with a partner. Of the total sample, 68% were employed, 9% were unemployed, and the remaining 23% were outside the labour force. One year after becoming parental caregivers, respondents reported lower fertility expectations compared to one year prior to becoming parental caregivers. Specifically, we observe an average decline of 0.8 in the fertility expectation score, which decreased from 7.8 to 7.0. In comparison with parents, childless adult children were, on average, 3.1 years younger, less likely to be in a union, and more likely to be employed. One year after becoming parental caregivers, childless respondents experienced a decline in the fertility expectation score of 0.7, whereas parents experienced a decline of 1.1. Figure 1 illustrates how the demands of caring for aging parents increase with age, with the percentage of adult children providing parental care gradually rising over the course of the reproductive life, from 1.1% at age 18–24 to 5.8% at age 45–49.

**Table 1:** Descriptive statistics of adult children’s characteristics (pooled analytical sample). *Source*: HILDA, waves 6–21, release 21

	(1)		(2)		(3)	
	Whole sample		Childless		Parents	
	Mean	SD	Mean	SD	Mean	SD
Age	29.58	7.33	28.56	7.17	31.68	7.24
Female	0.51	0.50	0.48	0.50	0.56	0.50
In a union	0.53	0.50	0.38	0.49	0.77	0.42
Employed	0.68	0.46	0.74	0.44	0.60	0.49
Unemployed	0.09	0.28	0.10	0.30	0.07	0.26
Out of LF	0.23	0.42	0.16	0.37	0.33	0.47
Fertility expectations before	7.79	1.97	7.85	1.92	7.67	2.07
Fertility expectations after	6.96	2.81	7.17	2.63	6.57	3.07
*N*	2643		1645		995	

*Fertility Expectations Before* refers to the average fertility expectations measured the year before the caregiving event. *Fertility Expectations After* refers to the average fertility expectations measured the year after the caregiving event

### Empirical Strategy

This paper aims to identify the causal impact of parental caregiving on fertility expectations. A naïve correlational analysis of parental caregiving and fertility expectations will likely be plagued by endogeneity and selection concerns. For example, individuals who become parental caregivers may be inherently different from individuals who do not, and, therefore, comparing individuals who are parental caregivers to individuals who are not would provide estimates that cannot credibly be given a causal interpretation. Along the same lines, comparing fertility expectations of individuals who become caregivers before and after they become caregivers would also not provide credible causal estimates, as, for example, fertility expectations tend to decline with age (Gray et al., 2013), regardless of whether individuals become parental caregivers.

The ideal experiment for identifying the short- and medium-run effects of parental caregiving on fertility expectations would randomly assign parental caregiving duties to individuals and track the evolution of their fertility expectations over time. This would allow us to compare the responses of those affected by caregiving responsibilities to the hypothetical behavior of similar, unaffected individuals. The access to a long panel of Australian survey data allows us to use a quasi-experimental research design to mimic this ideal experiment by taking advantage of the potential randomness of the timing when individuals become caregivers for their parents.

More in detail, we rely on a generalized difference-in-differences design, which compares trends over time for individuals who become parental caregivers at a certain point in our sample period to trends among contemporaneous “control” individuals who are observably similar but become parental caregivers shortly after.

It is important to note that the generalized difference-in-differences design, unlike a standard difference-in-differences design where all individuals in the treatment group are “treated” (i.e., become parental caregivers) at the same time (as in Lazzari et al., 2023), allows for the more general case where different individuals are treated at different times (“differential timing” or “staggered” design, see Roth et al., 2023 for details).

As a baseline specification, we therefore estimate the following staggered two-way fixed effect (TWFE) model, widely employed in the economics literature (Angrist & Krueger, 1999; Blundell & MaCurdy, 1999):

1 $$y_{{{\text{iat}}}} = \alpha _{i} + \delta _{{{\text{at}}}} + \sum\limits_{{l = b}}^{c} {\beta _{l} } D_{{{\text{it}}}}^{l} + X_{{{\text{it}}}}^{\prime } \gamma + \varepsilon _{{{\text{iat}}}}$$

where $y_{{{\text{iat}}}}$ denote the childbearing intentions of individual $i$ at time $t$, ${\alpha }_{i}$ are individual-level fixed effects and $\delta _{{{\text{at}}}}$ are age-by-calendar time fixed effects, which capture common shocks to childbearing intentions by age and calendar time. Time-varying characteristics such as employment status, relationship status, and parity are captured by $X_{{{\text{it}}}}$. Standard errors are clustered at the individual level. In all our event-study specifications, we normalize ${\beta }_{-1}=0$ and set $b=-5$ and $c=5$.

The event-study indicators $D_{{{\text{it}}}}^{l}$ are our treatment of interest, as they capture time from becoming a parental caregiver. Specifically, letting ${t}_{i}^{*}$ denote the time at which individual $i$, becomes a caregiver, we have that $D_{{{\text{it}}}}^{l} = 1\left\{ {t = t_{i}^{*} + l} \right\}$. The coefficients ${\beta }_{l}$ for $l\ge 0$ capture the effect of becoming a caregiver on childbearing intentions. Identification of ${\beta }_{l}$ hinges, first, on the assumption that individuals who have not become parental caregivers yet form a useful counterfactual for individuals who currently are parental caregivers. In other words, the assumption states that, absent the caregiving event, the outcomes of the treatment and control groups would have evolved parallely.

For the control group, comprising those who will experience a caregiving event in the future, to be valid, we require that both groups exhibit parallel trends before the event, after adjusting for age-by-calendar-year fixed effects and individual controls. It is not necessary for the two groups to be comparable in levels, only in trends.

**Fig. 2:**
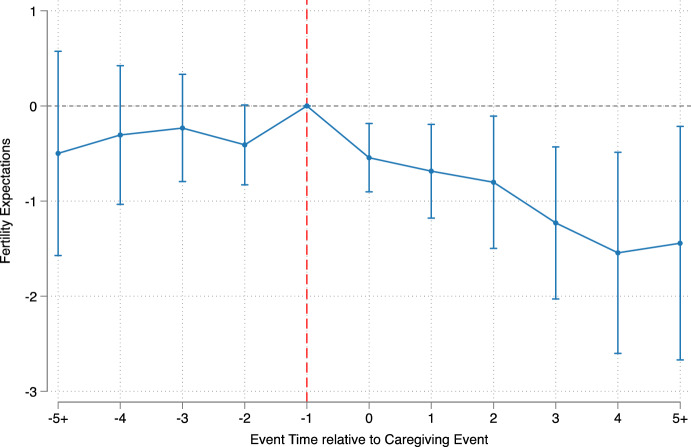
Effect of caregiving responsibilities on adult children’s fertility expectations. Coefficients and confidence intervals based on the linear fixed effects estimations of Model (1), with explanatory variables being: partnership status, employment, parity, survey year, and age. Source: HILDA, waves 6–21, release 21

This parallel-trend assumption can be assessed visually by evaluating the coefficients ${\beta }_{l}$ for $l<0$ in Fig. 2. If these coefficients are significantly different from zero, it would indicate that we cannot distinguish between the true effect of becoming parental caregivers and prior unobserved dynamics. Reassuringly, we observe that the coefficients for $l<0$ are not significant and close to zero, providing supporting evidence to the plausibility of the parallel trends assumption. Moreover, this result provides evidence of the suitability of our control group as counterfactual of the treatment group, suggesting that our method performs well even without using matching estimators, which might generate less precise estimates. Additionally, the absence of a response before the caregiving event suggests there are no anticipation effects.

The second identification assumption of the model is that average treatment effects are homogeneous across treated individuals and over time. Assuming these assumptions hold, the coefficients of interest ${\beta }_{l}$ for $l\ge 0$ identify the average treatment effect on the treated of parental caregiving on fertility expectations.

Under the assumptions above, the TWFE model allows us to rule out concerns that could otherwise limit our ability to interpret the estimates as causal. First, we can rule out that the results are driven by time-invariant differences in fertility expectations across individuals by including individual fixed effects. Second, we can rule out that our results are driven by fertility expectations evolving over time in a way that is common across all individuals. For example, the fact that, over time, the individuals in our panel are getting older might influence all individuals’ fertility expectations similarly. Age-by-calendar time fixed effects allow us to rule out such concerns.

Although TWFE regressions like Eq. (1) are commonly used for differential timing research designs, it has been shown that they deliver consistent estimates only under relatively strong assumptions about homogeneity in treatment effects (De Chaisemartin & d’Haultfoeuille, 2020; Borusyak et al., 2021; Callaway & Sant’Anna, 2021; Goodman-Bacon, 2021; Sun & Abraham, 2021). Specifically, if treatment effects are homogeneous across treated groups and across time, the TWFE estimator is consistent for the average treatment effect. Conversely, if treatment effects are heterogeneous across groups or time, the TWFE estimator does not deliver consistent estimates for the average treatment effect.

While our preferred specification does not include never-treated units (i.e., individuals who never become parental caregivers in our sample period), we address concerns about the reliability of TWFE estimator first by replicating our results including never-treated units in our estimation sample (Table S1 in Appendix). Second, we check the robustness of our estimates using the robust estimator introduced by Callaway and Sant’Anna (2021) (Figure S1 in Appendix). By shutting down the 2 × 2 difference-in-differences comparisons between newly treated and already treated units, the robust estimator delivers consistent estimates even in the presence of heterogeneous treatment effects across time and/or treated units. Given that the effect of a constraint on fertility expectations may differ depending on parity status, the analyses are conducted separately for parents and childless respondents.

## Results

Figure 2 displays point estimates and their 95% confidence intervals obtained by fitting Eq. (1). The x-axis reports the “event time”, as defined in the previous section, and captures the relative time from becoming a parental caregiver. The dashed gray line indicates where we would expect our coefficients to be if the effect of becoming a parental caregiver on fertility expectations was zero. The figure illustrates that in the immediate years after becoming a caregiver, individuals’ expectations about having children significantly decrease. The reduction in the fertility expectations score ranges from 0.5 to 1.5 per year. This effect intensifies over time, with an increased likelihood of not expecting to have children three years after the caregiving event. It is also important to note that the event-study coefficients are relatively flat and close to zero in the years prior to becoming a caregiver, providing suggestive evidence in favor of the parallel-trend assumption and, hence, credibility to our identification strategy.

Table 2 presents the regression results using Eq. (1) stepwise. To investigate heterogeneity in the timing of the effect of parental caregiving on fertility expectations, we present both the short-run effect (year 1 and 2 after the caregiving event) and the medium-run effect (from year 3 onwards), following the characterization in Fadlon and Nielsen (2021). In the fully adjusted model (column 4), becoming a caregiver has an estimated negative impact on fertility expectations of 7.3% in the short-run relative to the fertility expectations at baseline, while a greater reduction of 20% is estimated in the medium run. While a downward revision of fertility expectations with age is natural, the TWFE strategy implies that the drop in fertility expectations would have been less deep in the absence of the caregiving event. This estimated effect did not significantly vary by gender (Table S2 in Appendix).

**Table 2:** Effect of caregiving responsibilities on adult children’s fertility expectations. *Source*: HILDA, waves 6–21, release 21

	(1)	(2)	(3)	(4)
	Childbearing intentions	Childbearing intentions	Childbearing intentions	Childbearing intentions
treat*post SR	− 0.366*	− 0.379**	− 0.484***	− 0.460***
	(0.188)	(0.187)	(0.180)	(0.170)
treat*post MR	− 1.354***	− 1.479***	− 1.351***	− 1.267***
	(0.312)	(0.303)	(0.286)	(0.265)
Observations	2643	2643	2640	2640
R-squared	0.558	0.575	0.616	0.672
Ind. FE	Yes	YES	YES	YES
Year FE	Yes	YES	YES	YES
Age FE	No	YES	YES	YES
Controls	NO	NO	YES	YES
Age-by-Year FE	NO	NO	NO	YES

Standard errors clustered at the individual level in parentheses

treat*post SR refers to the interaction between a dummy equal to one if the individual became a caregiver at any point in the sample period and a dummy equal to one for the two periods after the caregiving event. treat*post MR refers to the interaction between a dummy equal to one if the individual ever became a caregiver and a dummy equal to one after period three after the caregiving event. Controls include partnership status, employment, and parity

***p < 0.01, **p < 0.05, *p < 0.1

In order to examine the potential variations in the impact of parental caregiving on fertility expectations, we conducted an analysis stratified by parity, as presented in Table 3. Our findings indicate that the negative effect of caregiving on fertility expectations does not exhibit a significant difference between parents and childless respondents in the first two periods following the event. However, after three years from the caregiving event, we observe a significantly more pronounced decline in fertility expectations among parents who have one child. We also investigate whether the results are heterogenous by socioeconomic class. For example, individuals from higher social classes might be less impacted by a caregiving shock since they may have the resources to obtain help with their caregiving duties. To analyze this potential variation, we examine heterogeneity by education level, which we use as an (imperfect) proxy for social classes. The results are reported in Table S6 and show that caregiving shocks impact fertility intentions similarly across socioeconomic classes.

**Table 3:** Effect of caregiving responsibilities on adult children’s fertility expectations: heterogeneity by parity. *Source*: HILDA, waves 6–21, release 21

	(1)
Variables	Childbearing intentions
treat*post SR	− 0.439**
	(0.186)
treat*post SR*Parity1	− 0.0384
	(0.357)
treat*post SR*Parity2 +	− 0.135
	(0.467)
treat*post MR	− 1.089***
	(0.302)
treat*post MR*Parity1	− 0.759*
	(0.428)
treat*post MR*Parity2 +	− 0.0454
	(0.589)
Observations	2640
R-squared	0.673
Ind. FE	YES
Year FE	YES
Age FE	YES
Controls	YES
Age-by-Year FE	YES

Standard errors clustered at the individual level in parentheses

treat*post SR refers to the interaction between a dummy equal to one if the individual became a caregiver at any point in the sample period and a dummy equal to one for the two periods after the caregiving event. treat*post MR refers to the interaction between a dummy equal one if the individual ever became a caregiver and a dummy equal to one after period three after the caregiving event. Controls include partnership status, employment, and parity

***p < 0.01, **p < 0.05, *p < 0.1

### Robustness Checks

We perform several additional robustness checks. First, we include never-treated observations in the estimation of specification (1). Including never-treated units can be important as recent papers (Goodman-Bacon, 2021) have warned against the identification of average treatment effects from two-way fixed effects models in the presence of heterogeneity of treatment effects. Including never-treated units in the analysis permits us to identify the event-study coefficients in Eq. (1) by confronting treated individuals to not-yet-treated and never-treated individuals. Results are reported in Table S1 in Appendix. Including never-treated individuals does not substantially change our results. In particular, the effect on childbearing intentions in the medium run (from period three onward after the event) remains negative and statistically significant.

Secondly, as explained above, we also replicate our results using the estimator proposed by Callaway and Sant’Anna (2021) that is robust to treatment effect heterogeneity. The results are reported in Figure S1, which shows that, independently of the estimator used, the coefficients before the caregiving event are close to zero and exhibit no discernible pre-trends. Moreover, independent of the estimator used, parental caregiving negatively and significantly impact fertility expectations.

Third, we re-run specification (1) including a control for individual health status. Controlling for health status can be important since previous research has shown that individuals adjust their childbearing desires and expectations in response to changes in their health conditions (Lazzari & Beaujouan, forthcoming). Table S3 in Appendix shows that our main results are robust to including health status as a control. Becoming a parental caregiver has a negative impact on fertility expectations both in the short and medium run. However, after controlling for health, the short-run coefficient is no longer statistically significant. In the medium run, we still find a negative and significant effect of parental caregiving on fertility intentions. Both coefficients are of similar magnitude to our preferred specification (column 4 of Table 1).

We also test the robustness of our results to restricting the sample to individuals who, prior to becoming caregivers, provided a score of 6, instead of 4, or higher on the fertility expectations scale. Table S4 reports the results which, reassuringly, are really similar to our baseline specification.

As an additional check to ensure that our main results are not driven by age, we tested for heterogeneity by age, dividing the sample into individuals younger and older than 30 years. The results, presented in Table S5, indicate that our main findings are not solely driven by older individuals, demonstrating that our primary effect is not merely an age effect.

## Discussion

The postponement of childbearing to later stages of life has brought about a shift in the life course context within which individuals plan parenthood. Consequently, it is essential to identify new factors within this context that may influence decisions about having children, factors that were less relevant in the past when the parenthood project was achieved at younger ages. One such factor may be the increasing demand for caring from aging parents, which has prompted our study to examine whether and how parental caregiving responsibilities impact the fertility plans of adult children.

Our study demonstrates that becoming a caregiver to elderly parents or parents-in-law results in an immediate and substantial decline in the fertility expectations of adult children. Furthermore, this effect intensifies over time, with an average reduction of approximately 7% in fertility expectations observed within the first two years of becoming a caregiver, and a deeper reduction of 20% observed three years after the caregiving responsibility began.

Although women typically bear most of the burden of caring for both children and the elderly (Australian Bureau of Statistics, 2018; Labbas & Stanfors, 2023), our study did not find any significant gender differences in the impact of caregiving responsibilities on fertility expectations. One possible explanation for this pattern is that the deterioration in the health of an elderly parent can disrupt relationship dynamics within the family. For example, if one partner assumes the role of primary caregiver, their reduced availability in terms of time and energy may impact the spouse, resulting in a spillover effect that impacts both partners equally. The absence of gender effects also suggests the potential relevance of the loss of grandparental support as a relevant factor in the decline of fertility expectations upon assuming caregiving responsibilities. Indeed, while the loss of childcare responsibilities has a clear impact on both partners, the burden of caregiving tends to disproportionately fall upon women.

Additionally, we observed that the negative impact of caregiving on fertility expectations is more pronounced among parents who have only one child three years after the caregiving event. This could be attributed to the fact that parents with one child have a more concrete understanding of the demands and responsibilities associated with parenthood. They are more familiar with the challenges and responsibilities of parenting compared to childless, who may be more optimistic about the effort required. Consequently, they may anticipate that the added caregiving responsibilities will significantly disrupt their existing plans, leading to a greater decline in their fertility expectations compared to childless individuals. Another explanation for this pattern is that parents with one child may have a clearer timeline in mind for when they intend to have their next child. Typically, the length of interval between first and second births is between two to four years (Köppen, 2006). If caregiving responsibilities cause them to miss this opportune window, a close spacing of first and second birth is no longer possible and the likelihood of making the transition to parenthood again at a later age diminishes. It is also possible that childless individuals may simply be more persistent in their expectation to have a child compared to parents facing constraints. This aligns with literature showing that people with more children are more likely to abandon their fertility intentions over time (Beaujouan, 2022; Spéder & Kapitány, 2009).

In addition to our empirical findings, we also provide a theoretical contribution by emphasizing how the changing demographics of prospective grandparents may impact reproductive choices among individuals of childbearing age. The findings of this study contribute to a deeper understanding of how changing external circumstances can shape fertility expectations and underscore the importance of examining the role of family networks in this process. Specifically, our results highlight that while grandparents can provide an important source of support to their adult children in their own childbearing decisions (Aassve et al., 2012a; Harknett et al., 2014; Rutigliano, 2020), on the other hand their need of support may compete with their children’s plan to form a family. This phenomenon goes beyond the concept of the ‘sandwich generation’, which refers to adults who are simultaneously caring for young children and elderly parents (Hammer & Neal, 2008; Perrig-Chiello & Hopflinger, 2005). Instead, it highlights the trend and implications of individuals *planning* their childbearing around the pressing needs of their aging parents. Whether future generations of prospective parents will face increased caregiving responsibilities due to longer life expectancy and delayed fertility depends on whether old age becomes not only longer but also healthier. The growing availability of grandparents, owing to their extended lifespan, could become a crucial resource for their adult children if they remain healthy enough to support their grandchildren’s upbringing without needing assistance themselves.

While our analyses reveal a negative effect of parental caregiving on fertility expectations, the underlying mechanisms driving this phenomenon are not fully understood. These effects may be attributed to the loss of parental support in the form of childcare or to a reduction in the time available for planning parenthood due to increased caregiving responsibilities for elderly parents. Our analysis does not allow us to differentiate between these two mechanisms or determine if the observed effect is the result of a combination of both factors. Nonetheless, our results strongly suggest that the negative impact of caregiving responsibilities on fertility expectations is more significant than any potential positive effect. In other words, it appears unlikely that the adult children taking on the new caregiver role would experience an increased sense of urgency to have children as it has been observed in response to parental death (Rackin & Gibson-Davis, 2022).

Our study examines how parental caregiving impacts the fertility expectations of adult children within a specific country. Therefore, the direct generalization of our findings to other settings with differing formal care policies, support measures for at-home elderly caregiving, and distinct childcare systems may be limited. For example, the research conducted by Labbas and Stanfors (2023) illustrates that the psychological wellbeing of caregivers can vary based on the extent of public elder care available in the country. Hence, it is possible that in contexts where there is greater public support for elder care, the negative influence of caregiving on fertility expectations would be less severe.

## Conclusion

In the context of delayed childbearing and population aging, understanding the impact of caregiving responsibilities toward the elderly on fertility expectations is of paramount importance. While previous literature already documents that caregiving responsibilities toward elderly parents or parents-in-law entails significant financial, social, and psychological costs for the caregiver, we provide new evidence that they may also lead to a negative revision of fertility expectations among adult children. In addition, by considering the intergenerational exchange of support and the potentially competing demands on caregiving resources, this study provides a more nuanced understanding of how parents may influence the fertility trajectories of their adult children. These findings have important implications for policies aimed at supporting intended parents in their plans to form a family. They highlight the significant impact that caregiving responsibilities toward one’s parent can have on individuals’ decisions to revise their childbearing plans and suggest that interventions aimed at reducing the caregiver burden could provide an opportunity to positively influence fertility levels.

## Supplementary Information

Below is the link to the electronic supplementary material.

Supplementary file1 (DOCX 135 KB)

## Data Availability

The HILDA dataset is available to approved researchers from government, academic institutions and non-profit organizations and can be accessed through https://dataverse.ada.edu.au/dataverse/hilda
