# Supplementary material for: The Effect of Parental Caregiving on the Fertility Expectations of Adult Children
Source: Eur J Popul. 2024 Nov 27;40(1):35. doi: 10.1007/s10680-024-09724-4 (PMC11602892; doi:10.1007/s10680-024-09724-4)
Supplement: Supplementary file 1 — Supplementary file1 (DOCX 135 KB) [file 10680_2024_9724_MOESM1_ESM.docx]

**Supplementary material**

**Table S1.** Effect of caregiving responsibilities on adult children’s fertility expectations, supplementary analysis including never treated respondents

|  | (1) |
| --- | --- |
|  | Childbearing Intentions |
|  |  |
| treat*post SR | -0.0468 |
|  | (0.138) |
| treat*post MR | -0.358* |
|  | (0.190) |
|  |  |
| Observations | 61,028 |
| R-squared | 0.664 |
| Ind. FE | YES |
| Year FE | YES |
| Age FE | YES |
| Controls | YES |
| Age-by-Year FE | YES |

*Note*: Standard errors clustered at the individual level in parentheses. *** p<0.01, ** p<0.05, * p<0.1. *treat*post SR* refers to the interaction between a dummy equal to one if the individual became a caregiver at any point in the sample period and a dummy equal to one for the two periods after the caregiving event. *treat*post MR* refers to the interaction between a dummy equal one if the individual ever became a caregiver and a dummy equal to one after period three after the caregiving event. Controls include partnership status, employment, and parity. *Source*: HILDA, waves 6-21, release 21.

**Figure S1.** Effect of caregiving responsibilities on adult children’s fertility expectations, supplementary analysis using Callaway and Sant’Anna Robust Estimator


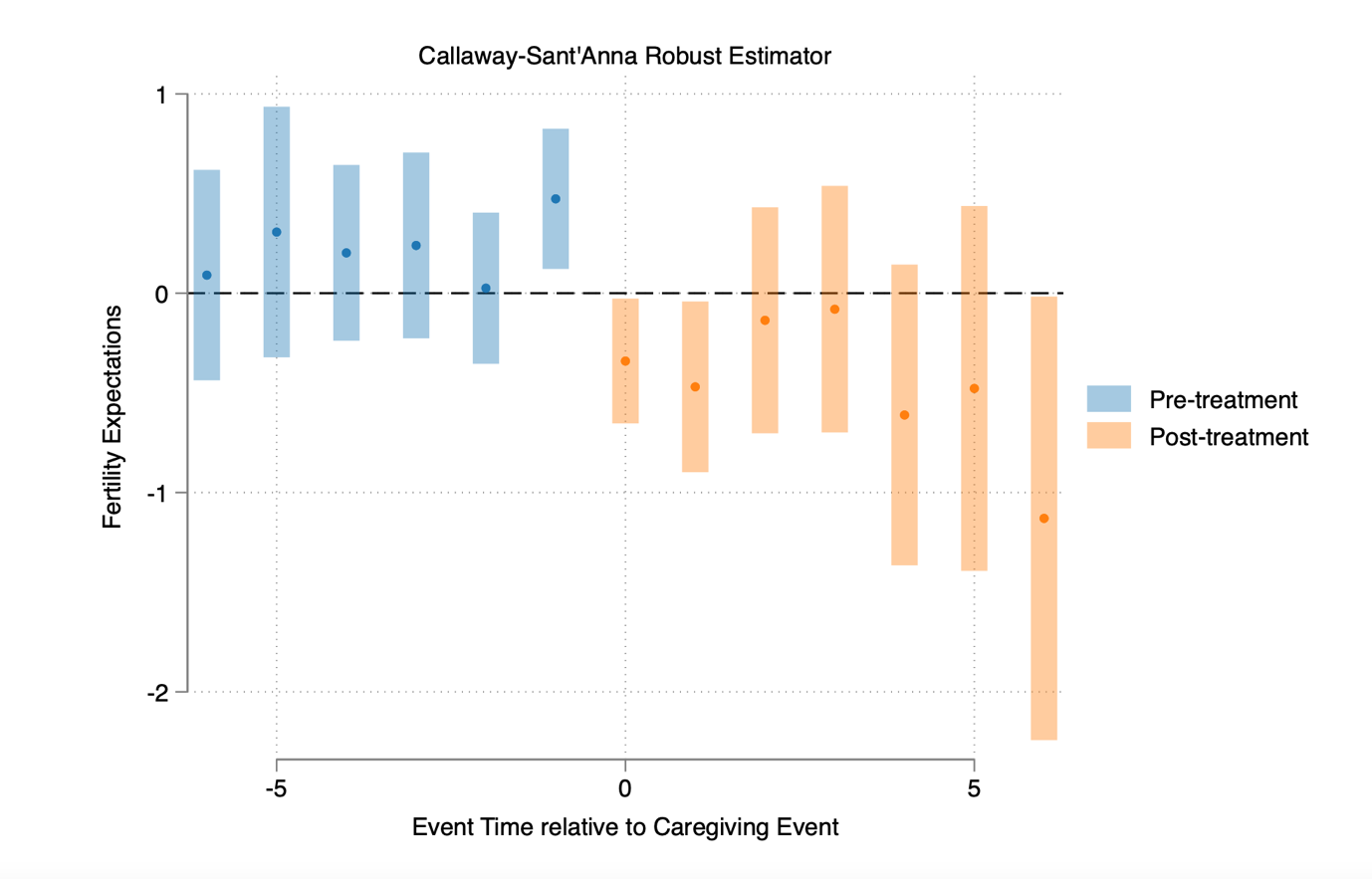


*Note*: Coefficients and confidence intervals based on TWFE estimation of Model (1) using the Callaway-Sant’Anna (2021) robust estimator, with explanatory variables being: partnership status, employment, parity, survey year, and age. *Source*: HILDA, waves 6-21, release 21.

**Table S2**. Effect of caregiving responsibilities on adult children’s fertility expectations: heterogeneity by gender

|  | (1) |
| --- | --- |
| VARIABLES | Childbearing Intentions |
|  |  |
| treat*post SR | -0.458** |
|  | (0.217) |
| treat*post SR*Female | -0.00369 |
|  | (0.294) |
| treat*post MR | -1.226*** |
|  | (0.324) |
| treat*post MR*Female | -0.0801 |
|  | (0.380) |
| Observations | 2,640 |
| R-squared | 0.672 |
| Ind. FE | YES |
| Year FE | YES |
| Age FE | YES |
| Controls | YES |
| Age-by-Year FE | YES |

*Note*: Standard errors clustered at the individual level in parentheses. *** p<0.01, ** p<0.05, * p<0.1. Controls include partnership status, employment, and parity. *Source*: HILDA, waves 6-21, release 21.

**Table S3.** Effect of caregiving responsibilities on adult children’s fertility expectations, supplementary analysis controlling for the health status of respondents

|  | (1) |
| --- | --- |
|  | Childbearing Intentions |
|  |  |
| treat*post SR | -0.303 |
|  | (0.188) |
| treat*post MR | -1.079*** |
|  | (0.295) |
|  |  |
| Observations | 2,220 |
| R-squared | 0.699 |
| Ind. FE | YES |
| Year FE | YES |
| Age FE | YES |
| Controls | YES |
| Age-by-Year FE | YES |
| Health | YES |

*Note*: Standard errors clustered at the individual level in parentheses. *** p<0.01, ** p<0.05, * p<0.1. Controls include partnership status, employment, and parity. *Source*: HILDA, waves 6-21, release 21.

**Table S4.** Effect of caregiving responsibilities on adult children’s fertility expectations, supplementary analysis using a different threshold (6 not 4)

|  | (1) | (2) | (3) | (4) |
| --- | --- | --- | --- | --- |
|  | Childbearing Intentions | Childbearing Intentions | Childbearing Intentions | Childbearing Intentions |
|  |  |  |  |  |
| treat*post SR | -0.484** | -0.515** | -0.660*** | -0.573*** |
|  | (0.220) | (0.214) | (0.205) | (0.208) |
| treat*post MR | -1.536*** | -1.734*** | -1.730*** | -1.623*** |
|  | (0.358) | (0.344) | (0.319) | (0.305) |
|  |  |  |  |  |
| Observations | 2,085 | 2,085 | 2,082 | 2,082 |
| R-squared | 0.575 | 0.593 | 0.631 | 0.706 |
| Ind. FE | YES | YES | YES | YES |
| Year FE | YES | YES | YES | YES |
| Age FE | NO | YES | YES | YES |
| Controls | NO | NO | YES | YES |
| Age-by-Year FE | NO | NO | NO | YES |

*Note*: Standard errors clustered at the individual level in parentheses. *** p<0.01, ** p<0.05, * p<0.1. Controls include partnership status, employment, and parity. *Source*: HILDA, waves 6-21, release 21.

**Table S5.** Effect of caregiving responsibilities on adult children’s fertility expectations, supplementary analysis to show heterogeneity by age (younger and older than 30 years old)

|  | (1) | (2) |
| --- | --- | --- |
|  | <30 | 30+ |
|  |  |  |
| treat*post SR | -0.423* | -0.538** |
|  | (0.231) | (0.264) |
| treat*post MR | -0.840** | -1.484*** |
|  | (0.372) | (0.429) |
|  |  |  |
| Observations | 1,275 | 1,319 |
| R-squared | 0.644 | 0.727 |
| Ind. FE | YES | YES |
| Year FE | YES | YES |
| Age FE | YES | YES |
| Controls | YES | YES |
| Age-by-Year FE | YES | YES |

*Note*: Standard errors clustered at the individual level in parentheses. *** p<0.01, ** p<0.05, * p<0.1. Controls include partnership status, employment, gender, and parity. *Source*: HILDA, waves 6-21, release 21.

**Table S6.** Effect of caregiving responsibilities on adult children’s fertility expectations, supplementary analysis to show heterogeneity by education level (less than high school diploma and with a high school diploma or more)

|  | (1) | (2) |
| --- | --- | --- |
| VARIABLES | Low Education | High Education |
|  |  |  |
| treat*post SR | -0.731** | -0.387* |
|  | (0.292) | (0.231) |
| treat*post MR | -1.168** | -1.393*** |
|  | (0.485) | (0.417) |
|  |  |  |
| Observations | 1,105 | 1,503 |
| R-squared | 0.749 | 0.734 |
| Ind. FE | YES | YES |
| Year FE | YES | YES |
| Age FE | YES | YES |
| Controls | YES | YES |
| Age-by-Year FE | YES | YES |

*Note*: Standard errors clustered at the individual level in parentheses. *** p<0.01, ** p<0.05, * p<0.1. Controls include partnership status, employment, gender, and parity. *Source*: HILDA, waves 6-21, release 21.
